# Cathelicidins Have Direct Antiviral Activity against Respiratory Syncytial Virus In Vitro and Protective Function In Vivo in Mice and Humans

**DOI:** 10.4049/jimmunol.1502478

**Published:** 2016-02-12

**Authors:** Silke M. Currie, Emily Gwyer Findlay, Amanda J. McFarlane, Paul M. Fitch, Bettina Böttcher, Nick Colegrave, Allan Paras, Agnieszka Jozwik, Christopher Chiu, Jürgen Schwarze, Donald J. Davidson

**Affiliations:** * Medical Research Council Centre for Inflammation Research, Queen’s Medical Research Institute, The University of Edinburgh, Edinburgh EH16 4TJ, United Kingdom;; † Institute for Quantitative Biology, Biochemistry and Biotechnology, School of Biological Sciences, The University of Edinburgh, Edinburgh EH9 3BF, United Kingdom;; ‡ Institute of Evolutionary Biology, School of Biological Sciences, The University of Edinburgh, Edinburgh EH9 3BF, United Kingdom; and; § National Heart and Lung Institute, Imperial College London, London W2 1PG, United Kingdom

## Abstract

Respiratory syncytial virus (RSV) is a leading cause of respiratory tract infection in infants, causing significant morbidity and mortality. No vaccine or specific, effective treatment is currently available. A more complete understanding of the key components of effective host response to RSV and novel preventative and therapeutic interventions are urgently required. Cathelicidins are host defense peptides, expressed in the inflamed lung, with key microbicidal and modulatory roles in innate host defense against infection. In this article, we demonstrate that the human cathelicidin LL-37 mediates an antiviral effect on RSV by inducing direct damage to the viral envelope, disrupting viral particles and decreasing virus binding to, and infection of, human epithelial cells in vitro. In addition, exogenously applied LL-37 is protective against RSV-mediated disease in vivo, in a murine model of pulmonary RSV infection, demonstrating maximal efficacy when applied concomitantly with virus. Furthermore, endogenous murine cathelicidin, induced by infection, has a fundamental role in protection against disease in vivo postinfection with RSV. Finally, higher nasal levels of LL-37 are associated with protection in a healthy human adult RSV infection model. These data lead us to propose that cathelicidins are a key, nonredundant component of host defense against pulmonary infection with RSV, functioning as a first point of contact antiviral shield and having additional later-phase roles in minimizing the severity of disease outcome. Consequently, cathelicidins represent an inducible target for preventative strategies against RSV infection and may inform the design of novel therapeutic analogs for use in established infection.

## Introduction

Respiratory syncytial virus (RSV) is an important pathogen of the human respiratory tract (1). RSV infection results in viral bronchiolitis in ∼30% of infants who become infected, and it can result in life-threatening severe bronchiolitis and viral pneumonia in ∼2% of all infants (2). RSV causes significant mortality in the developing world, resulting in an estimated 200,000 annual deaths in young children globally, in addition to major morbidity (33.8 million episodes worldwide annually) (3). Although the majority of children recover after only mild symptoms, children experiencing severe or recurrent bronchiolitis have an increased risk for recurrent wheeze and asthma (4, 5). The variability in susceptibility to RSV-induced disease and outcomes is not understood and is proposed to have host- and virus-specific causes, as well as showing seasonal variation. In addition, apart from costly passive immunization, which is reserved for very high risk infants, there is no vaccine or effective specific treatment available for RSV bronchiolitis, other than supportive measures (6). Therefore, a clearer understanding of components of host defense that contribute to effective protection against RSV infection and disease is urgently required and could inform the development of novel preventative or therapeutic strategies.

We have previously demonstrated that the human cathelicidin LL-37 has dose-dependent antiviral activity against RSV in vitro (7). Cathelicidins are a family of host defense peptides (also known as antimicrobial peptides), with key functions in the innate immune system, having both direct microbicidal and multiple host defense modulatory functions (reviewed in Ref. 8). These peptides are expressed over a broad range of sites in infection and inflammation, generated primarily by neutrophils and epithelial cells (reviewed in Refs. 9, 10). Humans and mice each encode only one cathelicidin; the human cationic antimicrobial peptide of 18 kDa (hCAP-18) is the sole human cathelicidin, encoded by the *CAMP* gene (11, 12), and the murine ortholog mCRAMP is encoded by the murine *Camp* gene (13). LL-37, the main active form of human cathelicidin, is generated proteolytically from hCAP-18 (14), can be detected in a wide range of body fluids, including airway surface liquid, and is upregulated by infection and inflammation (8). Our previous work has indicated that cathelicidin may represent an important targetable component of innate host defense against RSV infection (7). However, the mechanism of action of this peptide-mediated antiviral activity, the in vivo potential of exogenously applied cathelicidins, and the physiological significance of endogenous respiratory tract expression of cathelicidin in RSV infection and disease remained unknown.

In this article, we demonstrate that LL-37 mediates an antiviral effect on RSV via direct damage to the viral envelope, disrupting viral particles and decreasing virus binding to, and infection of, epithelial cells. This activity results in protection against RSV infection and disease in a murine model of pulmonary RSV infection, demonstrating maximal efficacy when LL-37 is applied concomitantly with virus. In addition, murine cathelicidin, mCRAMP, also has antiviral activity against RSV in vitro, is induced in the lungs in response to RSV, and contributes to protection against disease postinfection with RSV. Finally, higher nasal levels of LL-37 are associated with protection against infection in a healthy human adult RSV infection model.

## Materials and Methods

### Ethics statement

Mouse experiments were performed in accordance with Home Office UK project license 60/4216, under the Animal (Scientific Procedures) Act 1986. For human studies, a cohort of healthy nonsmoking adults (aged 18–33 y; with no underlying immunodeficiencies) was enrolled, providing written informed consent, approved by the UK National Research Ethics Service West London Research Ethics Committee (study numbers 10/H0711/94 and 11/LO/1826) and the Imperial College Joint Research Office.

### Peptide synthesis

LL-37, scrambled LL-37 (scrLL-37), and mCRAMP peptides were custom synthesized by Almac (East Lothian, U.K.) using Fmoc solid-phase synthesis and reversed phase HPLC purification. Peptide identity was confirmed by electrospray mass spectrometry, purity (>95% area) by reversed phase HPLC, and net peptide content determined by amino acid analysis. Lyophilized peptides were reconstituted in endotoxin-free water at 5 mg/ml stock concentration and determined to be endotoxin-free using a *Limulus* Amebocyte Lysate Chromogenic Endotoxin Quantitation Kit (Thermo Scientific). Peptide functionality was confirmed by assessing antiendotoxic activity (15).

### Cell culture

HEp-2 cells (ATCC CCL-23) were cultured in DMEM/F12 (Life Technologies/Life Technologies, Paisley, U.K.), supplemented with 2 mM l-glutamine (PAA Laboratories, Yeovil, U.K.) and 10% FCS (Biosera, East Sussex, U.K.), at 37°C and 5% CO_2_.

### Virus propagation and purification

RSV strain A2 was purchased from American Type Culture Collection (ATCC-VR1540). Virus was propagated by infecting 70% confluent HEp-2 cells with a multiplicity of infection (MOI) of 0.1 for 2 h in 6 ml serum-free DMEM-F12, after which 20 ml medium containing 10% FCS (PAA Laboratories) was added and cells were cultured for 24 h. FCS content was reduced to 2% and virus was harvested at 70% cytopathic effects. Supernatant was clarified by centrifugation, snap-frozen, and stored at −80°C. For sucrose purification, clarified supernatant was centrifuged through a 7.5-ml 30% (w/v) sucrose cushion (24,000 rpm, 2 h, 4°C, Beckman XI-90 ultracentrifuge, SW28 rotor), with brake set to minimum. Virus pellets obtained from each 175-cm^2^ cell culture flask were resuspended in 200 μl serum-free DMEM-F12. For envelope-preserving inactivation, sucrose-purified RSV-A2 was treated with 10 mM Aldrithiol (Sigma-Aldrich, Dorset, U.K.) for 24 h, 4°C, and was snap-frozen and stored at −80°C.

### LL-37 solid-phase ELISA

High-affinity binding plates [High bind microplate (Costar Corning, Tewksbury, MA) 3590 were coated with 1 × 10^6^ PFU RSV in coating buffer (15 mM Na_2_CO_3_, 35 mM NaHCO_3_, pH 9.6)], control cell culture medium, or 2.5% BSA for 12 h at 4°C, washed with PBS, and incubated with 2.5% BSA for 3 h. Wells were exposed to 2 μg/ml LL-37 for 30 min at 4°C, washed with 0.02% Tween 20/PBS. Bound LL-37 was detected by anti–LL-37 Ab (1:6400, G-075-06; Phoenix Pharmaceuticals, Burlingame, CA), HRP-labeled secondary Ab [1:5000, goat anti-rabbit IgG (H+L) (Jackson Laboratories, West Grove, PA), 111-035-045], and TMB substrate. Reaction was stopped using 0.5 M sulfuric acid, and absorbance at A450 was determined using a plate reader (Synergy HT; Biotek, Winooski, VT).

### RSV solid-phase ELISA

High-affinity binding plates were coated with 2 μg/ml LL-37 or scrLL-37 for 12 h at 4°C, washed with PBS, and incubated with 2.5% BSA for 3 h. RSV 3.25 × 10^5^ PFU was added for 30 min at 4°C, and wells were washed with 0.02% Tween 20/PBS. For detection of bound RSV, wells were exposed to anti-RSV Ab (1:200; Goat Anti Respiratory Syncytial Virus Biotin Polyclonal 7950-0104; AbD Serotec, Kidlington, U.K.), HRP-labeled secondary Ab (1:10,000, P0449; Dako, Glostrup, Denmark), and TMB substrate (Bio-Rad). Reaction was stopped using 0.5 M sulfuric acid, and absorbance at A450 was determined using a plate reader (Synergy HT; Biotek, Winooski, VT).

### Immunofluorescence staining for N/F-protein colocalization by confocal microscopy

As described by Krzyzaniak et al. (16), 5 × 10^2^ PFU RSV was exposed to 25 μg/ml LL-37, scrLL-37, or endotoxin-free water, immediately air-dried on glass slides and fixed (10% neutral buffered formalin; Sigma-Aldrich). Particles were permeabilized with 0.2% Triton X-100 (Sigma-Aldrich) and incubated 1% BSA in TBST (1 h, 4°C; Sigma-Aldrich). F- and N-protein were detected using primary Abs [1:100 Anti-Respiratory Syncytial Virus Ab (2F7), ab43812 (Abcam, Cambridge, U.K.), and Anti-RSV Ab, nucleoprotein, clone 130-12H, MAB858-3 (Millipore, Darmstadt, Germany) respectively], followed by Alexa Fluor–labeled secondary Abs [1:50, Alexa Fluor 488 Goat-anti mouse IgG2a (y2a), Alexa Fluor594 Goat-anti mouse IgG1 (Y1); Life Technologies]. Samples were embedded in ProLong Diamond Antifade reagent (Life Technologies). Slides were viewed with a Leica SP confocal microscope (original magnification ×100), collecting 10 images per treatment condition. Colocalization was analyzed using IMARIS software (Bitplane, Zurich, Switzerland), with thresholds set to include particles >0.05 μm and signal quality higher than 15, and particles were counted using ImageJ.

### Transmission electron cryomicroscopy

A total of 4 × 10^4^ PFU Aldrithiol-inactivated RSV was exposed to 25 μg/ml LL-37 or endotoxin-free water. Then, 4 μl pretreated RSV was applied to freshly glow-discharged Quantifoil-grids (R2/2, 400 mesh; Quantifoil, Großlöbichau, Germany) and vitrified with a FEI Vitrobot IV (FEI, Hillsboro, OR) at 100% humidity and 4°C (blot force 10, blot time 2 s). Vitrified samples were transferred with a Gatan 626 cryoholder into a FEI-F20 electron microscope operated at 200 kV. Micrographs were semiautomatically acquired with EM-tools (TVIPS) on an 8kx8k CMOS detector (Tvips F816) at a nominal original magnification ×50,000 (calibrated pixel size, 1.53 Å/Px) and with an approximate dose of 20 e^−^/Å^2^.

### Assessment of IFN-β, IL-28, and IL-29 expression by quantitative RT-PCR

HEp-2 cells were infected with RSV (MOI = 1) for 2 h, inoculum was replaced by fresh medium, and infection continued for 24 h. Cells were lysed and RNA was isolated using the Qiagen RNeasy mini kit (Manchester, U.K.) according to manufacturer’s instructions. cDNA was generated by means of quantitative RT-PCR (Q-RT-PCR) using the TaqMan Reverse Transcription Reagents (Applied Biosystems, Bleiswijk, the Netherlands) according to manufacturer’s instructions (25°C: 10 min, 48°C: 40 min, 95°C: 5 min, 4°C: hold). TaqMan-based Q-RT-PCR was performed for human *IFN-β* (Life Technologies, Paisley, U.K.) and human *IL-28* and *IL-29* (sequences kindly provided by Dr. Michael Edwards, Imperial College London). VIC-labeled 18S primer/probe mix (Applied Biosystems) was used as endogenous control. Samples were run on an ABI Prism 7900HT Real-Time PCR System. The primer sequences used included: human *IL-28AB*: forward, 5′-CTGCCACATAGCCCAGTTCA-3′; reverse, 5′-AGAAGCGACTCTTCTAAGGCATCTT-3′; probe, 5′-TCTCCACAGGAGCTGCAGGCCTTTA-3′; human *IL-29*: forward, 5′ GGACGCCTTGGAAGAGTCACT-3′; and reverse: 5′-AGAAGCCTCAGGTCCCAATTC-3′; probe, 5′-AGTTGCAGCTCTCCTGTCTTCCCCG-3′.

### Quantification of RSV binding by flow cytometry and Western blotting

Following a protocol adapted from Krzyzaniak et al. (16), HEp-2 cells were detached using versene solution (Sigma Aldrich), washed, exposed to 25 μg/ml LL-37, scrLL-37, or endotoxin-free water, and chilled on ice for 30 min. Cells were washed and exposed to RSV (MOI = 1) concomitant with 25 μg/ml LL-37, scrLL-37, or endotoxin-free water for 1 h at 4°C. For flow cytometry, cells were washed, fixed (4% paraformaldehyde; Sigma-Aldrich), resuspended in anti–F-protein Ab [1:200, Anti-Respiratory Syncytial Virus Ab (2F7), ab43812; Abcam] for 12 h at 4°C, and stained with AF488-labeled secondary Ab [1:200, Alexa Fluor 488 goat anti-mouse IgG2a (y2a); Life Technologies]. Samples were run on the FACSCalibur (BD Biosciences, Oxford, U.K.) using Cell Quest (BD Biosciences) V3.2.1 within 1 h, and results were analyzed using FlowJo (FlowJo, Ashland, OR).

For Western blotting, RSV-exposed cells were washed, taken up in sample buffer and reducing agent (Invitrogen, Paisley, U.K.), and incubated at 95°C for 10 min. SDS-PAGE on 10% Bis/Tris gels in MES buffer (Invitrogen) was performed, protein was transferred to nitrocellulose membranes, which were exposed to anti–F-protein Ab (1:200, Anti-Respiratory Syncytial Virus Ab [2F7], ab43812; Abcam), followed by HRP-labeled secondary Ab (goat anti-mouse, 115-035-062; Jackson Laboratories). Chemiluminescence (Amersham ECL Prime Western Blotting Detection Reagent; GE Healthcare, Little Chalfont, U.K.) was detected by x-ray film, and band density was quantified using ImageJ.

### In vitro antiviral activity of mCRAMP by immunoplaque assay

Confluent HEp-2 cells were infected with RSV (MOI = 0.005) concomitant with 1, 10, 25, and 50 μg/ml mCRAMP, 25 μg/ml LL-37, or endotoxin-free water for 2 h at 37°C. Inoculum was removed, infection was continued for 24 h at 37°C, and cells were fixed (2% hydrogen peroxide in methanol). Cells were exposed to biotinylated anti-RSV Ab (1:200, Goat Anti Respiratory Syncytial Virus Biotin Polyclonal, 7950-0104; AbD Serotec, Oxford, U.K.), washed, incubated with ExtrAvidin peroxidase (1:500; Sigma), and washed again. 3-Amino-ethylcarbazole substrate was used according to manufacturer’s instructions (Sigma-Aldrich). Positive immunoplaques were quantified by light microscopy.

### Infection and peptide treatments of mice

For experiments using wild-type mice, female BALB/c mice were supplied by Charles River Laboratories (Tranent, U.K.), maintained with littermates in local specified pathogen-free facilities in individually ventilated cages for at least 1 wk before experimental use at 6–8 wk of age. Mice were randomly allocated into infection or control cages with matched littermates. Within each cage, mice were randomly allocated to different treatment groups (LL-37 or scrLL-37, and/or varied timing of peptide delivery dependent upon the study), with only one mouse per condition per cage, the mouse being the experimental unit and repeated experimental blocks conducted on different days. Mice received 100 μl RSV (5.6 × 10^5^ PFU) or PBS by intranasal delivery under light general anesthesia (isoflurane). A total of 10 μg/mouse LL-37 or scrLL-37 (given as 100 μl of 100 μg/ml solution) was either given 1 h before infection, or coadministered or given 1 h postinfection. Mice further received a daily dose of 10 μg/mouse LL-37 or scrLL-37 until day 6 postinfection. Body weights were monitored daily, and groups of mice were culled on day 4 or 7 postinfection. For studies using cathelicidin-deficient mice, wild type C57BL/6J OlaHsd and *Camp^−/−^* mice (17), backcrossed to congenicity into C57BL/6J OlaHsd strain in-house, were bred from homozygous matings in-house in specified pathogen-free facilities, in individually ventilated cages. Female sister pairs of both genotypes (8–12 wk) were randomly partnered and cohoused as one wild-type and one *Camp^−/−^* mouse per cage, with the pair of cages allocated as one infected and one control. Mice received 2.3–5.6 × 10^5^ PFU RSV or PBS by intranasal delivery under light general anesthesia (isoflurane), body weights were monitored daily, and mice were culled on days 4 and 6 postinfection. Lungs were removed and processed as described later.

### Assessment of mCRAMP protein expression by Western blotting

Lung tissue was homogenized [Precellys CK14 (Kit ceramic, 1.4 mm, 2-ml tubes, 432-3751, settings 5000-2x50, Precellys24 tissue homogenizer; Bertin Technologies] in MPer (Thermo Fisher Scientific, Paisley, U.K.), lysed under constant agitation (4°C, 20 min), and supernatant collected. Protein content of lysed tissue supernatant was determined using a bicinchoninic acid assay (Pierce BCA Protein Assay Kit; Thermo Scientific), according to manufacturer’s instructions, to enable equivalent loading. Samples were incubated at 95°C for 10 min in sample buffer and reducing agent [NuPAGE LDS Sample buffer (4×) and NuPAGE Sample Reducing Agent (10×); Invitrogen]. SDS-PAGE on 4–12% Bis/Tris gels in MES buffer (Invitrogen) was performed, protein was transferred to nitrocellulose (Nitrocellulose Membrane Filter paper, Sandwich, 0.2-μm pore size; Life Technologies), and membranes were exposed to anti-mCRAMP Ab (1:500, CRAMP Ab, PA-CPPL-100; Innovagen AB, Lund, Sweden) and anti–β-actin Ab (1:20000, clone AC-15; Sigma-Aldrich), followed by the respective IRDye680LT or 800CW Li-Cor labeled secondary Ab (Li-Cor Biotechnology, Cambridge, U.K.). Fluorescence intensity was quantified using a Li-Cor Biosciences Odyssey 9120 Infrared Imaging System.

### Assessment of Camp transcription and viral load by Q-RT-PCR

Lung tissue was homogenized in cell lysis buffer (20 mM Hepes, 0.3 mM NaCl, 1.5 mM, MgCl_2_, 0.2 mM EDTA, 1 mM DTT, 1 mM orthovanadate, 0.5% Triton X-100, Complete Protease Inhibitor Cocktail Tablets; Roche Diagnostics, Basel, Switzerland), supernatant was isolated, and RNA was prepared using the Qiagen RNeasy mini kit according to manufacturer’s instructions. cDNA was generated using TaqMan Reverse Transcription Reagents (Applied Biosystems) according to manufacturer’s instructions (25°C: 10 min, 48°C: 40 min, 95°C: 5 min, 4°C: hold). Q-RT-PCR (TaqMan Gene Expression Master Mix; Applied Biosystems) for RSV *L-gene* was performed on an ABI Prism 7900HT Real-Time PCR System, either using a commercial primer/probe set [murine, Mm00438285_m1, TaqMan (Applied Biosystems); VIC-labeled 18S primer/probe mix was used as an endogenous control], or using the following primers and probe set: RSV *L gene*: Fwd, 5′-GAACTCAGTGTAGGTAGAATGTTTGCA-3′; Rev, 5′−TTTCAGCTATCATTTTCTCTGCCAAT-3′; probe, 5′−TTTGAACCTGTCTGAACATTCCCGGTT-3′.

### Histology

For a representative subset of mice, left lung lobes were fixed in 10% neutral buffered formalin (4°C, 12 h) and then transferred to 70% ethanol and processed to sections by SuRF@QMRI (The University of Edinburgh). Samples were inserted into tissue tek cassettes, dehydrated, and embedded in paraffin wax using a LEICA ASP processor and sectioned. Sections were baked for 12 h at 55°C, followed by dewaxing in xylene and rehydration using descending concentrations of ethanol baths (100 to 70%). Sections were stained with hematoxylin (5 min; Sigma-Aldrich), washed, and differentiated in 1% acid alcohol, before slides were transferred to Scott’s tap water substitute (Sigma-Aldrich) and transferred to eosin solution (2 min; Thermo Fisher Scientific). Slides were washed, dehydrated, and cleared using rising concentrations of ethanol baths (70–100%) and xylene. Lung sections were assessed for severity of inflammation, blinded to genotype and treatment.

### Experimental RSV infection of human adults

A cohort of healthy nonsmoking adults (aged 18–33 y; with no underlying immunodeficiencies) was enrolled, and infection studies were conducted as previously described (18). In brief, nasal lavage (collected by instilling 0.9% saline using a 10-ml syringe with attached metal nasal olive [Clinical Engineering, Royal Brompton Hospital, London, U.K.] and washing the nose 10 times over a 1-min period) and blood samples were collected immediately before inoculation with 10^4^ PFU RSV A Memphis 37 [M37 (Meridian Lifesciences, Memphis, TN); a low-passage virulent strain of RSV that was produced according to Good Manufacturing Practice) by intranasal drops. Bronchoalveolar lavage was obtained 2 wk before inoculation. Subjects were subsequently housed in residential quarantine for 10 d. Infection was defined by detection of RSV nucleoprotein gene by Q-RT-PCR in nasal lavage on at least 2 d between days 2 and 10 postinoculation. Samples were stored at −80°C and quantified for hCAP-18/LL-37 by ELISA (HK321-02; Hycult Biotech, Uden, the Netherlands) according to manufacturer’s instructions, using samples as collected (i.e., not concentrating the lung and nasal washes). Study remained blinded until completion.

### Statistical analysis

Statistical analysis was performed using the GraphPad PRISM5 statistical package (GraphPad Software, La Jolla, CA) and Minitab 16 statistical software (Minitab, Coventry, U.K.) by Student *t* test, one-way ANOVA with Dunnett multiple comparison posttest, two-way ANOVA with Bonferroni posttest, or general linear model ANOVA as stated in the respective figure legends. The *p* values <0.05 were considered significant.

## Results

### LL-37 binds directly to RSV virions

**FIGURE 1.**
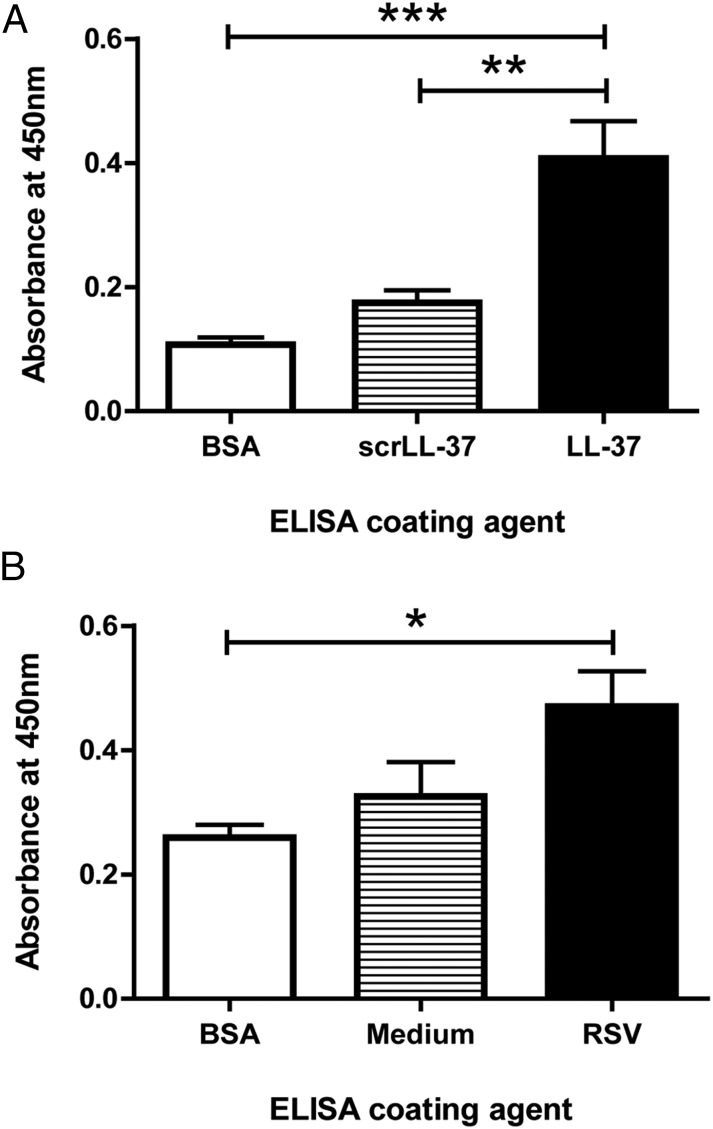
LL-37 binds directly to RSV. LL-37 (**A**) and RSV (**B**) solid-phase ELISAs were performed by coating high-affinity binding plates with BSA, RSV, or cell culture medium (A) or BSA, LL-37, or scrLL-37 (B). Plates were incubated with LL-37 (A) or RSV (B), and were either exposed to anti–LL-37 or anti-RSV Ab, followed by HRP-labeled secondary Abs and detection with TMB. Data show mean absorbance at 450 nm from *n* = 3 independent experiments, each performed in triplicate, and analyzed by one-way ANOVA with Dunnett posttest (*p < 0.05, **p < 0.01, ***p < 0.001).

To determine whether LL-37 directly interacts with RSV, we assessed peptide binding to virion particles by ELISA. To guarantee specificity of the assay, we tested binding in both directions. Wells coated with LL-37 (or BSA, or scrLL-37 as controls) were incubated with RSV, followed by detection of binding with anti-RSV Ab (Fig. 1A). RSV had a significantly higher binding affinity to LL-37 than to BSA or to scrLL-37. Wells coated with RSV (or, as binding controls, coated with either BSA or medium derived from uninfected cells) were incubated with LL-37 peptide, followed by detection of binding using anti–LL-37 Ab (Fig. 1B). LL-37 showed significantly greater binding to RSV than to BSA control. In contrast, LL-37 binding to tissue culture medium–coated wells was equivalent to BSA-coated wells. These data demonstrate that LL-37 directly binds RSV virion particles.

### LL-37 disrupts the RSV viral membrane

**FIGURE 2.**
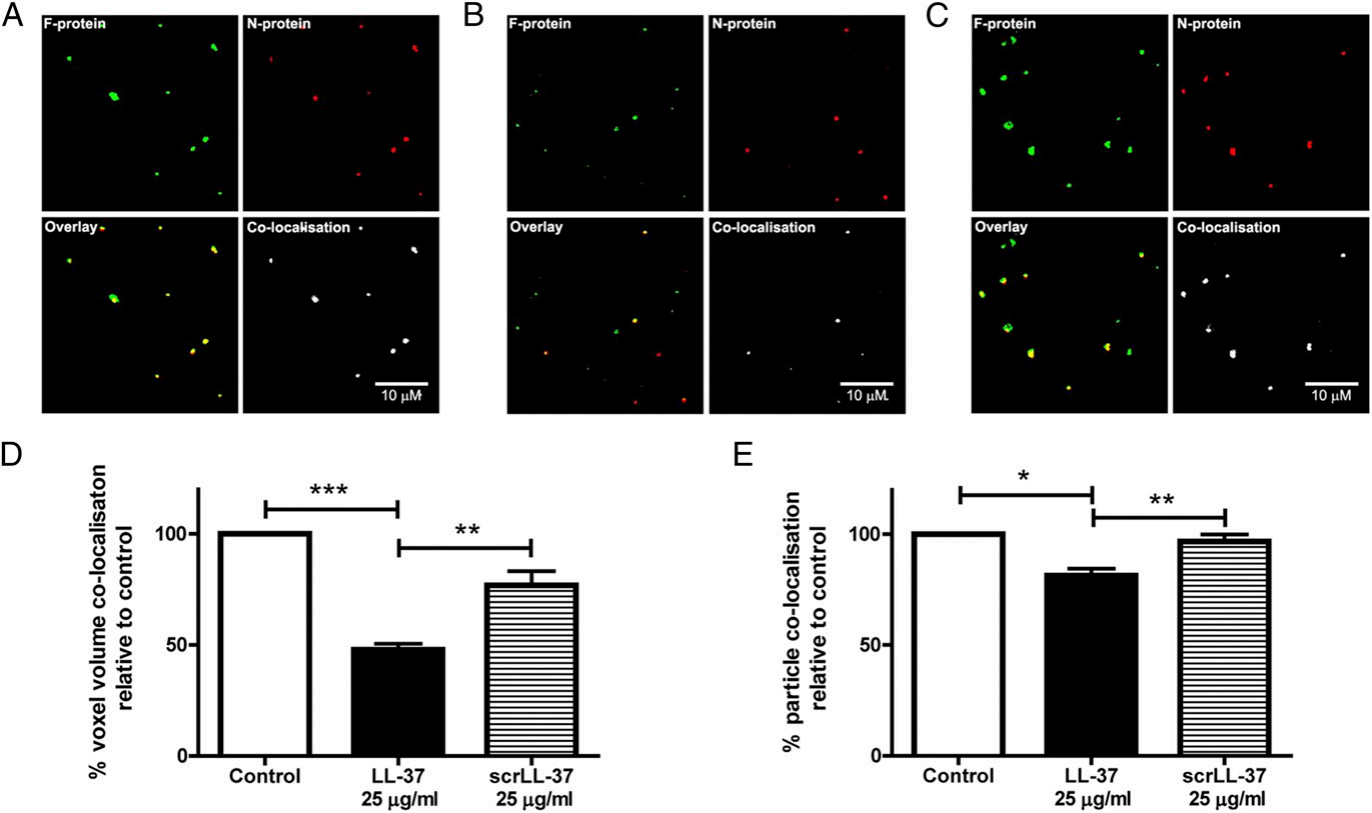
Exposure to LL-37 reduces RSV N- and F-protein colocalization. RSV, treated with endotoxin-free water (as vehicle control; **A**), 25 μg/ml LL-37 (**B**), or 25 μg/ml scrLL-37 (**C**), was immediately air-dried on glass slides, fixed, and permeabilized, before incubation with anti-RSV F- and N-protein Abs, followed by labeled secondary Abs and assessment by confocal microscopy. (A–C) Images show representative regions (original magnification ×100) demonstrating F-protein (F AF488; green), N-protein (N AF594; red), overlay of channels, and completely colocalizing particles as determined by the IMARIS. The volume of fully colocalized signal quantified using IMARIS (**D**) and the number of double-positive particles counted using ImageJ (**E**) is displayed as a percentage, relative to untreated virus. Data represent mean ± SEM for *n* = 3 independent experiments, performed in duplicate, assessing at least 10 random fields per condition (100–500 particles per field of view), analyzed by one-way ANOVA with Dunnett posttest (*p < 0.05, **p < 0.01, ***p < 0.001).

The functional conformation of viral envelope-bound proteins, including attachment- and fusion-proteins, is preserved by the integrity of the viral envelope (19). Changes in its structural integrity can lead to altered protein folding, causing loss of function. To investigate whether LL-37 binding of RSV caused destabilization of viral particles, we examined immunofluorescent colocalization of the RSV fusion protein (F-protein; which is bound on the viral envelope) and N-protein (found in the capsid), as previously described (16). Confocal microscopic assessment of untreated, purified RSV samples for F- and N-protein revealed three distinct populations (Fig. 2A): intact viral particles (colocalization of F-and N-proteins), capsid-free virus-like particles (F-protein only), and free capsid material (N-protein only). Changes in the proportions of each population after treatment with LL-37 (Fig. 2B) or scrLL-37 (Fig. 2C) were then quantified (Fig. 2D, 2E) by IMARIS. RSV exposed to LL-37 demonstrated a significantly lower overall level of colocalization of the F- and N-proteins (representing partial and/or complete viral particle disruption) than controls and scrLL-37–treated virus (Fig. 2D). Furthermore, there was significantly more complete particle disruption (demonstrated by single signal-positive particles) and a corresponding decrease in the number of particles with any degree of colocalized F- and N-signals (Fig. 2E). These data demonstrate LL-37–mediated separation of envelope- and capsid-based viral proteins.

**FIGURE 3.**
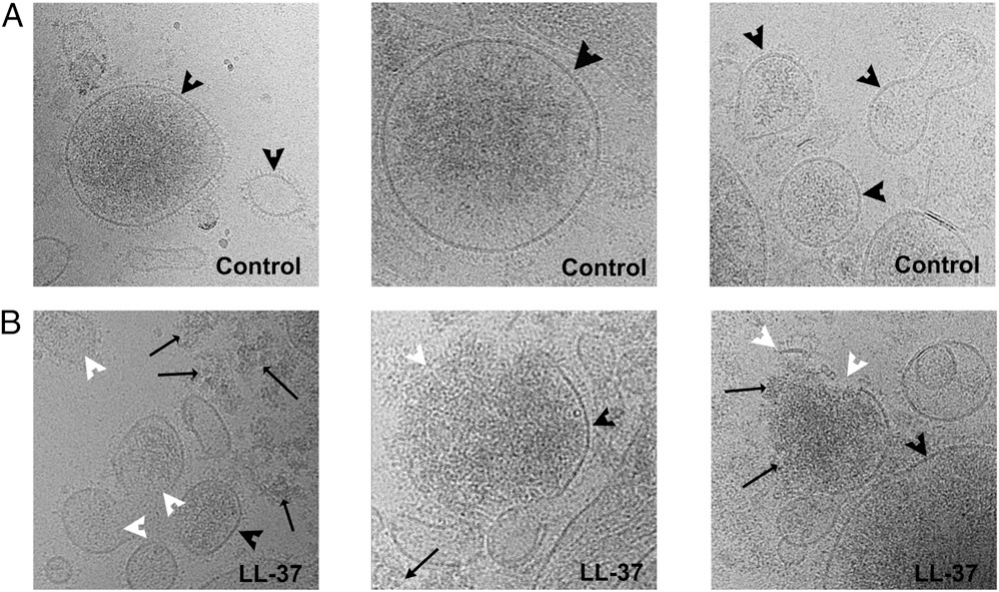
Exposure to LL-37 induces damage to RSV membrane integrity. Cryotransmission electron micrographs of control RSV (**A**) and RSV exposed to 25 μg/ml LL-37, followed by immediate vitrification (**B**). Micrographs were taken at a nominal original magnification ×50,000 and underwent visual inspection using ImageJ. Micrographs represent observations from at least two independent experiments, with a minimum of two grids analyzed per condition. Black arrowhead indicates intact membrane; white arrowhead indicates membrane damage; and black arrow indicates free capsid material.

To visualize the impact of LL-37 on RSV viral membrane, we assessed envelope integrity by transmission electron cryomicroscopy after exposure to LL-37 (Fig. 3) and vitrification, which preserves the physiological state of envelope structures (20). Untreated, purified RSV virions, with a range of shapes and sizes, showed continuous envelope lining with the capsid contained within the virus, in keeping with previous observations (21). In contrast, in LL-37–exposed RSV samples, more particles with obviously disrupted envelopes and free capsid content were observed. Taken together, these data demonstrate a direct interaction between LL-37 and RSV, resulting in breakdown of envelope integrity and viral particle disruption.

### LL-37 inhibits epithelial cell binding of RSV and responses to infection

**FIGURE 4.**
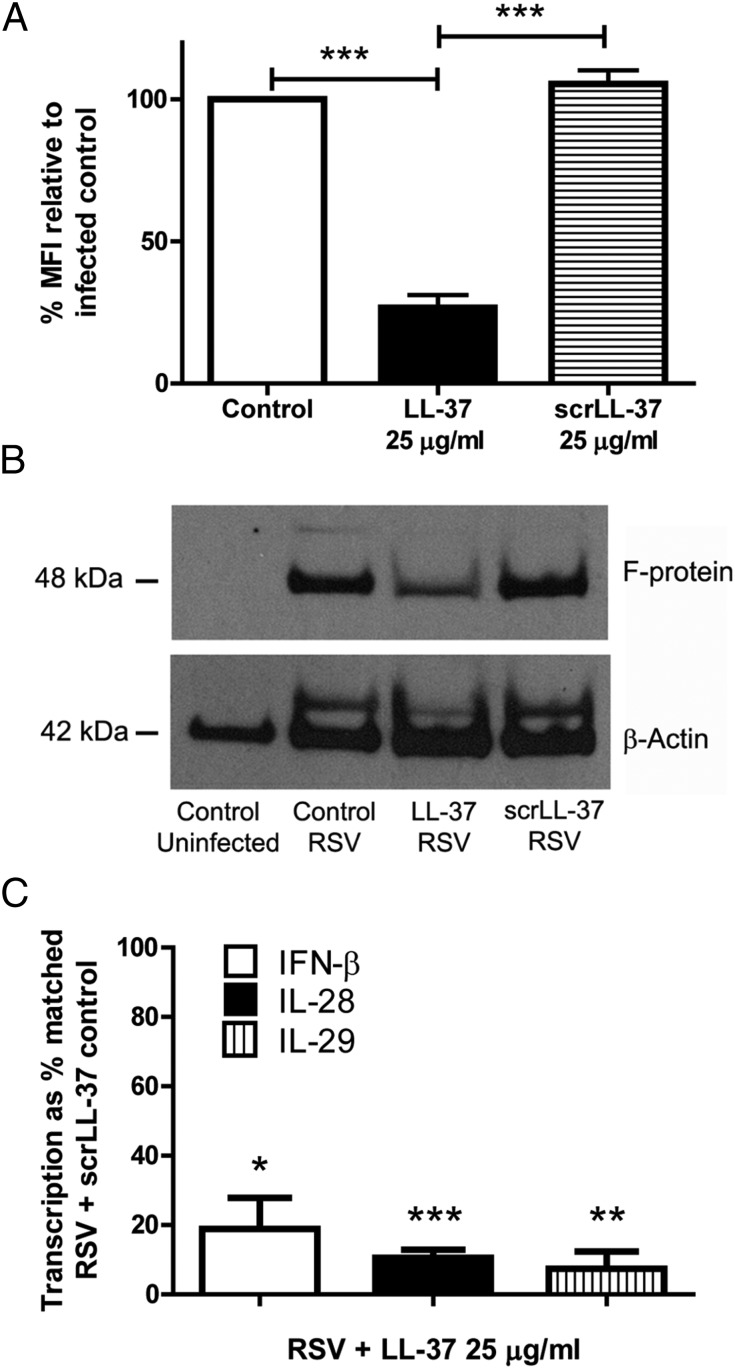
LL-37 exposure results in lower level of RSV binding to HEp-2 cells. HEp-2 cells were infected with RSV (MOI = 1) at 4°C (**A** and **B**) or 37°C (**C**), delivered concomitantly with 25 μg/ml LL-37, 25 μg/ml scrLL-37, or carrier control, for 1 (A and B) or 2 h (C). Cells were then either fixed, labeled with anti-RSV F-protein Ab and corresponding secondary Ab and analyzed by flow cytometry (A), protein collected and examined by Western immunoblot for F-protein and β-actin, as a loading control (B), or washed and incubated for a further 24 h before collection of total cell RNA for TaqMan-based Q-RT-PCR (C). (A) Mean fluorescence intensity (MFI) normalized to control infected cells for *n* = 3 independent experiments, performed in duplicate and analyzed by one-way ANOVA with Dunnett posttest (***p < 0.001). (B) Western blot for F-protein in total infected HEp-2 cell protein, representative of *n* = 3 independent experiments. (C) Transcription of *IFN-β*, *IL-28*, and *IL-29* in RSV-infected cells treated concomitantly with LL-37, as a percentage of the matched RSV-infected cells treated concomitantly with scrLL-37, showing mean ± SEM for *n* = 3 independent experiments, performed in duplicate.

Damage to the integrity of the RSV envelope could result in loss of function of envelope-bound attachment- and fusion-proteins, and thus impaired cellular binding (19). HEp-2 epithelial cells and reagents were cooled to 4°C to inhibit the active fusion process of RSV with epithelial cell membranes, while still permitting viral binding (22), and then exposed to RSV for 1 h at 4°C with concomitant exposure to LL-37, scrLL-37, or carrier only control. Viral binding was assessed by flow cytometric analysis of epithelial F-protein (Fig. 4A) and by F-protein Western blot of total protein collected (Fig. 4B). LL-37, given concomitantly with RSV infection, resulted in significantly lower levels of association of F-protein, and thus RSV, with HEp-2 cells, relative to the scrLL-37-treated and control infected cells. The scrambled control peptide had no effect on binding, in either assay.

To then assess the impact of LL-37 on the epithelial cell response to RSV infection at 37°C, we examined the transcription of type I and III IFN genes 24 h postinfection with concomitant administration of LL-37, or scrLL-37 control. Transcription of *IFN-β*, *IL-28*, and *IL-29* was induced by RSV infection in all samples but was significantly lower in LL-37–treated cells (Fig. 4C). Taken together, these results are compatible with the notion that exposure of RSV to LL-37, but not scrLL-37, at the time of infection results in damage to viral particles, decreased binding to epithelial cells, decreased infectivity, and subsequently, a lesser cellular response to the virus.

### LL-37 is protective against RSV infection in mice in vivo

Having demonstrated that LL-37 had antiviral properties against RSV in vitro, the question whether these properties were functional in vivo was addressed. A well-characterized model of murine pulmonary RSV infection was used (23), following progress of disease over 6 d after intranasal inoculation with RSV. Groups of four littermates were separated into infection and control caged pairs, with one mouse in each pair receiving LL-37 and the other receiving scrLL-37 intranasally, concomitant with RSV or carrier control. Mice were subsequently treated with daily intranasal inoculation of either LL-37 or scrLL-37. Weight loss was assessed daily, pulmonary transcription of *Ifn-β* was assessed on day 1, and viral load was determined on days 4 and 7.

**FIGURE 5.**
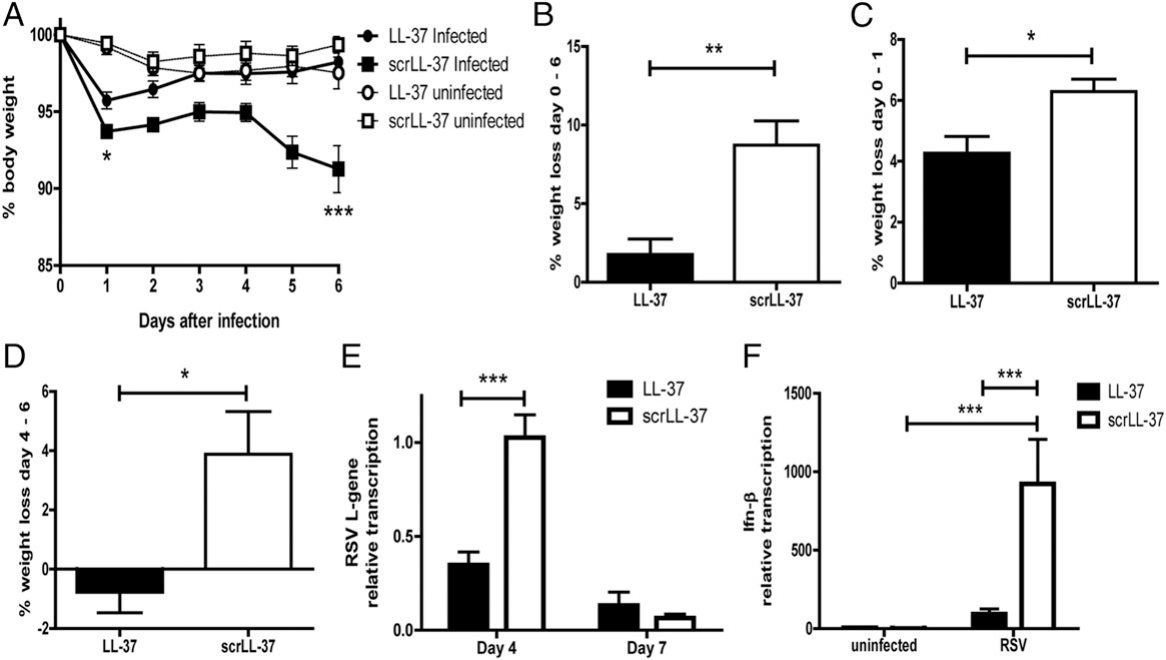
LL-37 is protective against RSV infection in vivo. Six- to eight-week-old female BALB/c mice in groups of four littermates were housed in infected and uninfected sister pair cages and intranasally inoculated with 3–6 × 10^5^ PFU RSV or PBS, concomitant with 10 μg LL-37 (given as 100 μl of 100 μg/ml solution) or scrLL-37 on day 0. On days 1–6, all mice received further inoculations of LL-37 or scrLL-37 intranasally, and weight was monitored and assessed as a percentage of starting weight. (**A**–**D**) Data represent *n* = 9 animals per group, performed in three independent experimental blocks, shown as mean ± SEM, analyzed by (A) general linear model ANOVA (***p < 0.001, *p < 0.05) and (B–D) Wilcoxon matched-pairs signed rank test (**p < 0.01, *p < 0.05). (**E**) Pulmonary RSV *l-gene* transcription was assessed at 4 and 7 d postinfection and expressed as fold change relative to the mean value for scrLL-37–treated infected mice at virus peak on day 4. Data show mean ± SEM from *n* = 6 mice/condition, analyzed by two-way ANOVA with Bonferroni posttest (***p < 0.0001). (**F**) Pulmonary *Ifn-β* expression was assessed on day 1 postinfection and expressed as fold change relative to the mean value for scrLL-37–treated uninfected. Data show mean ± SEM from *n* = 6 mice/condition, analyzed by two-way ANOVA with Bonferroni posttest (***p < 0.001).

RSV-infected mice treated with scrLL-37showed an early weight reduction on day 1 postinfection, and a later period of weight loss between days 4 and 6 (Fig. 5A), compatible with the established model for untreated, RSV-infected mice (24). In contrast, RSV-infected mice treated with LL-37 had significantly less weight loss over the entire course of the experiment, when compared with scrLL-37–treated infected controls (Fig. 5B), showing a lesser early weight loss (Fig. 5C) and no later weight loss (Fig. 5D), being indistinguishable from uninfected controls from day 3 onward (Fig. 5A). Using a general linearized model (to assess the efficacy of LL-37 against infection, relative to any impact of peptide treatment alone), we compared the difference in weight loss between matched infected and uninfected littermates given LL-37 with the difference between their infected and uninfected littermates given scrLL-37, and this was found to be significant at both the early (*p* < 0.05) and the later (*p* < 0.01) phases of weight loss, and for total weight loss over the whole study (*p* < 0.001). Analysis of RSV *L-gene* transcription in the lung (Fig. 5E) showed a significant, 60% lower viral load in the LL-37–treated mice compared with the scrLL-37–treated animals at day 4 (the time point of peak viral load in this model) (25). No difference between LL-37– and scrLL-37–treated mice was observed by day 7, by which time the virus has largely been cleared in this model. These data could be compatible with rapid early antiviral activity of LL-37 directly reducing the effective infective load of virus, LL-37 treatment promoting enhanced clearance of RSV, or LL-37 preventing spread of infection. To examine the magnitude of early host response to initial viral load and infection, we evaluated *Ifn-β* transcription at day 1 (Fig. 5F). Uninfected animals, treated daily with LL-37 or scrLL-37, did not show any substantial expression of *Ifn-β.* In contrast, control infected mice, treated with scrLL-37, had a large, significant induction of *Ifn-β* expression in response to virus. However, infected mice treated with LL-37 had significantly lower levels of *Ifn-β* expression than control infected animals, compatible with lower initial infectious load. These data support a rapid, early impact on RSV infectivity, resulting in protection against disease.

### Prophylactic or therapeutic intranasal delivery of LL-37 in murine RSV infection

**FIGURE 6.**
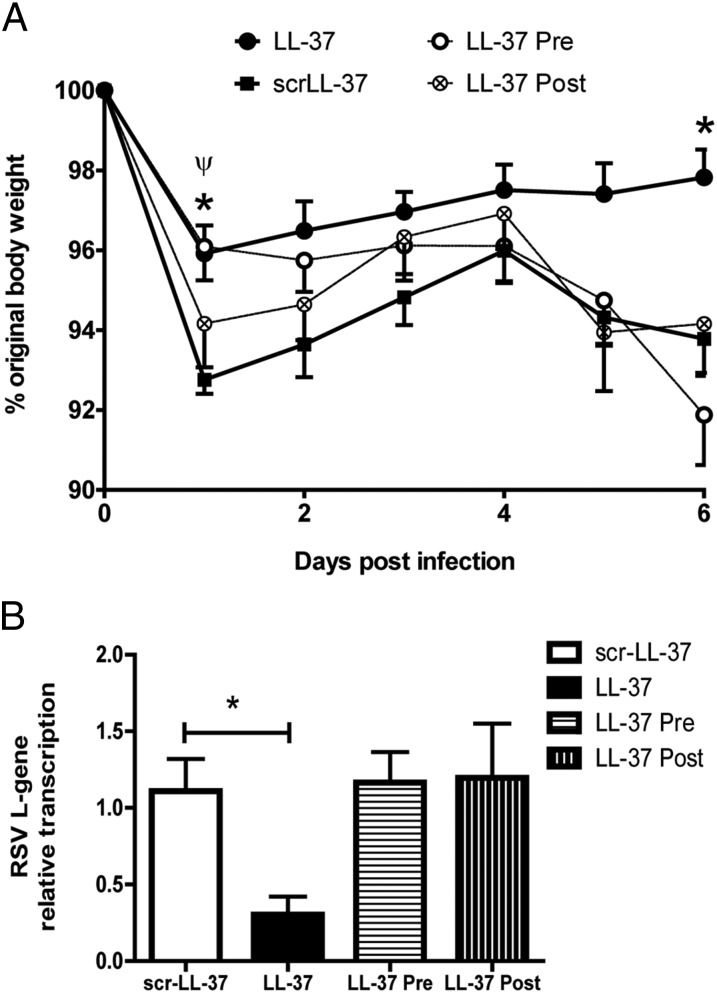
Prophylactic or therapeutic LL-37 administration in RSV infection. Six- to eight-week-old female BALB/c mice in groups of four cohoused littermates were intranasally inoculated on day 0 with 3–6 × 10^5^ PFU RSV and 10 μg LL-37 (given as 100 μl of 100 μg/ml solution), applied intranasally either 1 h before, 1 h after, or concomitantly with infection, or with concomitant delivery of scrLL-37. On days 1–6, all mice received further inoculations of LL-37 or scrLL-37 as appropriate, and weight was monitored and assessed as a percentage of starting weight. (**A**) Data represent *n* = 8 animals per group, performed in three independent experimental blocks, shown as mean ± SEM, analyzed by one-way ANOVA with Dunnett posttests, *p < 0.05 (comparing concomitant LL-37 with control infected), ^ψ^*p* < 0.05 (comparing LL-37 pretreatment with control infected). (**B**) Pulmonary RSV *L-gene* transcription was assessed at 4 d postinfection and expressed as fold change relative to the mean value for scrLL-37–treated infected mice. Data show mean ± SEM from *n* = 4–6 mice/condition, analyzed by one-way ANOVA with Dunnett posttests (*p < 0.05).

The exposure of epithelial cells to LL-37 before RSV infection resulted in reduced viral infection in vitro (7). In addition, LL-37 treatment applied after initiation of RSV infection prevented the spread of infection in vitro (7). Therefore, in vivo studies were conducted to determine whether the protective properties demonstrated with concomitant application of LL-37 could be extended to prophylactic and/or therapeutic potential against RSV infection in vivo. Groups of four littermate mice were intranasally inoculated with LL-37 either 1 h before, 1 h after, or concomitantly with RSV infection, or with concomitant scrLL-37 application, and subsequently treated with continued daily intranasal inoculation with either LL-37 or scrLL-37 as appropriate. In contrast with the significant protective effects of concomitant LL-37 delivery, neither pretreatment nor delayed initial application of LL-37 had protective effects on weight loss over the entire course of the infection (Fig. 6A), nor did these approaches alter viral load at 4 d postinfection (Fig. 6B). However, application of LL-37 at 1 h before virus did result in significantly less weight loss in the first 24 h postinfection, comparable with that of concomitant delivery of LL-37. These data suggest that effective pulmonary colocalization of RSV and LL-37 may be critical for immediate direct antiviral activity.

### mCRAMP has antiviral activity against RSV in vitro and is induced by RSV in vivo

**FIGURE 7.**
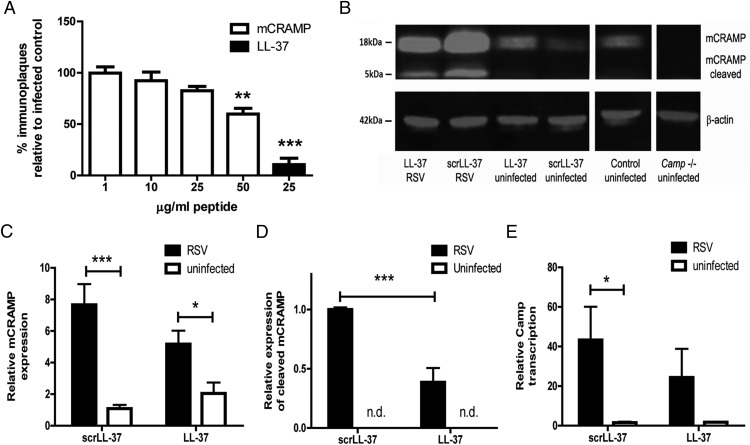
Murine cathelicidin is antiviral against RSV and induced by infection. (**A**) HEp-2 cells were infected with RSV administered concomitantly with a concentration range of mCRAMP (1, 10, 25, 50 μg/ml), 25 μg/ml LL-37, or carrier control for 2 h, washed, and incubated for 24 h in fresh medium. Immunoplaque assay was used to quantify the number of infected cells relative to the infected carrier control. Data show mean ± SEM for *n* = 3 independent experiments, performed in triplicate, analyzed by one-way ANOVA with Dunnett multiple comparison posttest (**p < 0.01, ***p < 0.001). (**B**–**D**) Wild type mice were intranasally inoculated with 3–6 × 10^5^ PFU RSV or PBS, concomitant with 10 μg LL-37 (given as 100 μl of 100 μg/ml solution) or scrLL-37, and culled 24 h later. Uninfected *Camp*^−/−^ mice were used as a negative control. Total protein and RNA were prepared from lung lobes and assessed for mCRAMP and β-actin (as a loading control) by Western immunoblot (B) quantified by Li-Cor Odyssey (C and D), and assessed for *Camp* expression by TaqMan-based Q-RT-PCR (**E**). Data show mean ± SEM expression levels, relative to the mean level in scr-LL-37–treated uninfected controls, from *n* = 6 per group, analyzed by two-way ANOVA with Bonferroni posttest (*p < 0.05, ***p < 0.0001). n.d., not detected.

Mice, like humans, express one sole cathelicidin (13). The murine ortholog of hCAP-18/LL-37 is mCRAMP, expressed by the *Camp* gene. To investigate whether mCRAMP also has antiviral activity against RSV, we infected HEp-2 cells in the presence or absence of mCRAMP over a range of concentrations. Concomitant application of the murine peptide mCRAMP was found to have a similar, concentration-dependent antiviral activity on RSV in this in vitro assay (Fig. 7A), but to be less potent than the human peptide LL-37. Having established that mCRAMP had anti-RSV activity in vitro, the extent to which the peptide was expressed in the murine lung in vivo was determined in uninfected mice and on day 1 postinfection with RSV, both in the presence and the absence of concomitant LL-37 treatment. The proform of mCRAMP, but not the cleaved active peptide, was detected by Western blot at low levels in the uninfected lung (Fig. 7B–D). Treatment with LL-37 or scrLL-37 alone had no significant effect on mCRAMP expression. However, at 1 d after RSV infection, *Camp* transcription (Fig. 7E) and mCRAMP peptide levels (Fig. 7C, 7D) were both significantly upregulated, and the active cleaved form was now detected (Fig. 7B). The quantity of cleaved mCRAMP detected was significantly greater in the control infected mice than the LL-37–treated infected animals (Fig. 7D), compatible with a larger effective infectious stimulus. These data suggest that endogenous cathelicidin could also play an antiviral role against RSV infection in the murine lung.

### Endogenous mCRAMP is protective in RSV infection in vivo

**FIGURE 8.**
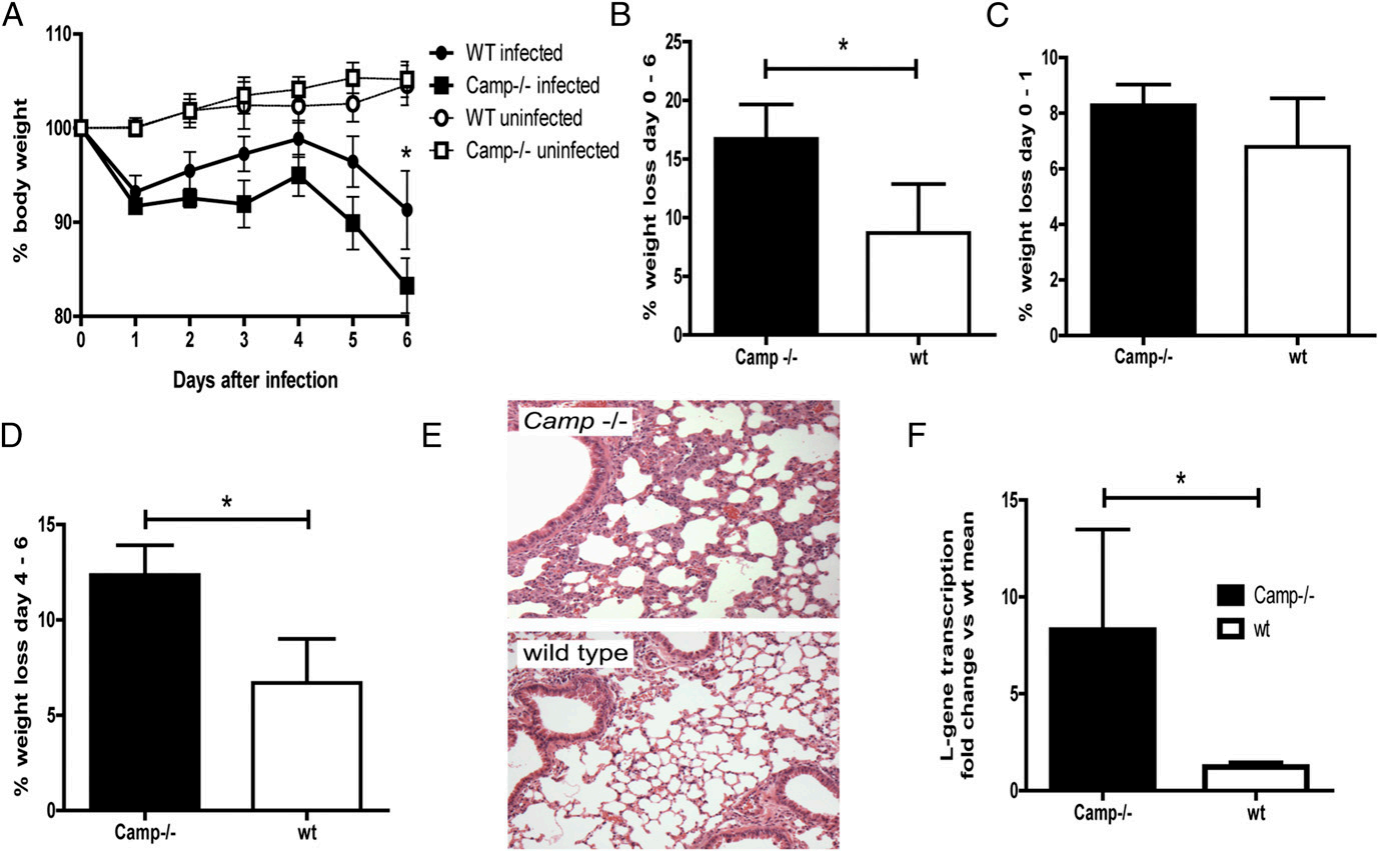
Endogenous mCRAMP is protective against RSV infection in vivo. Eight- to twelve-week-old female littermate pairs of *Camp^−/−^* or wild-type (wt) C57BL/6J OlaHsd mice were split between infected and matched uninfected cages, cohousing both genotypes. Mice were infected with 3–6 × 10^5^ PFU RSV. Weight was monitored and assessed as a percentage of starting weight. (**A**–**D**) Data represent *n* = 6 animals per group, performed in three independent experimental blocks, shown as mean ± SEM, analyzed by Wilcoxon matched-pairs signed rank test, comparing genotypes (*p < 0.01). (**E**) Light micrographic images of pulmonary histology of H&E-stained lungs collected at day 6 postinfection, shown at original magnification ×100, representative of *n* = 3 per genotype. (**F**) Pulmonary RSV *L-gene* transcription was assessed at 3 d postinfection and expressed as fold change relative to the mean value for wt infected mice. Data show mean ± SEM from *n* = 8 mice per condition, analyzed by Wilcoxon matched-pairs signed rank test.

Having established that mCRAMP has anti-RSV potential and is induced by RSV infection, the significance of this peptide was evaluated in *Camp^−/−^* mice, using the intranasal RSV infection model. Littermate pairs were separated into infected and uninfected cages, cohousing wild-type and *Camp^−/−^* mice in each cage, studied in a series of experimental blocks. Mice were inoculated on day 0 with RSV, or PBS as a carrier control. Weight loss was monitored for 6 d, viral load was assessed at day 3, and lung histology was examined at day 6. Uninfected mice of both genotypes gained weight over the course of the experiment (Fig. 8A). RSV-infected mice had two phases of weight loss, as described earlier, with the *Camp^−/−^* mice losing significantly more weight over the course of the study (Fig. 8A, 8B). In contrast with the effects of concomitant LL-37 inoculation, deletion of the *Camp* gene had no significant effect on weight loss over the first 24 h (Fig. 8C). This is a period in which the active, cleaved form of mCRAMP was not present in either genotype (Fig. 7B–D), but induction of *Camp* was initiated in wild type controls (Fig. 7E). However, weight loss in the second phase (days 4–6) was significantly greater in the *Camp^−/−^* mice, with these animals losing almost twice as much weight over that period than the paired wild-type controls (Fig. 8D), and by the end of the study, *Camp^−/−^* mice had more extensive inflammatory infiltrates in the lungs (Fig. 8E). This increased disease severity was accompanied by a greater viral load (assessed by *L-gene* expression) in the *Camp^−/−^* mice at day 3 postinfection (Fig. 8F), compared with matched wild type controls. These data demonstrate that endogenous production of mCRAMP during RSV infection has a nonredundant role in limiting disease severity.

### Susceptibility to human experimental RSV infection is associated with lower basal LL-37 levels in nasal lavage

**FIGURE 9.**
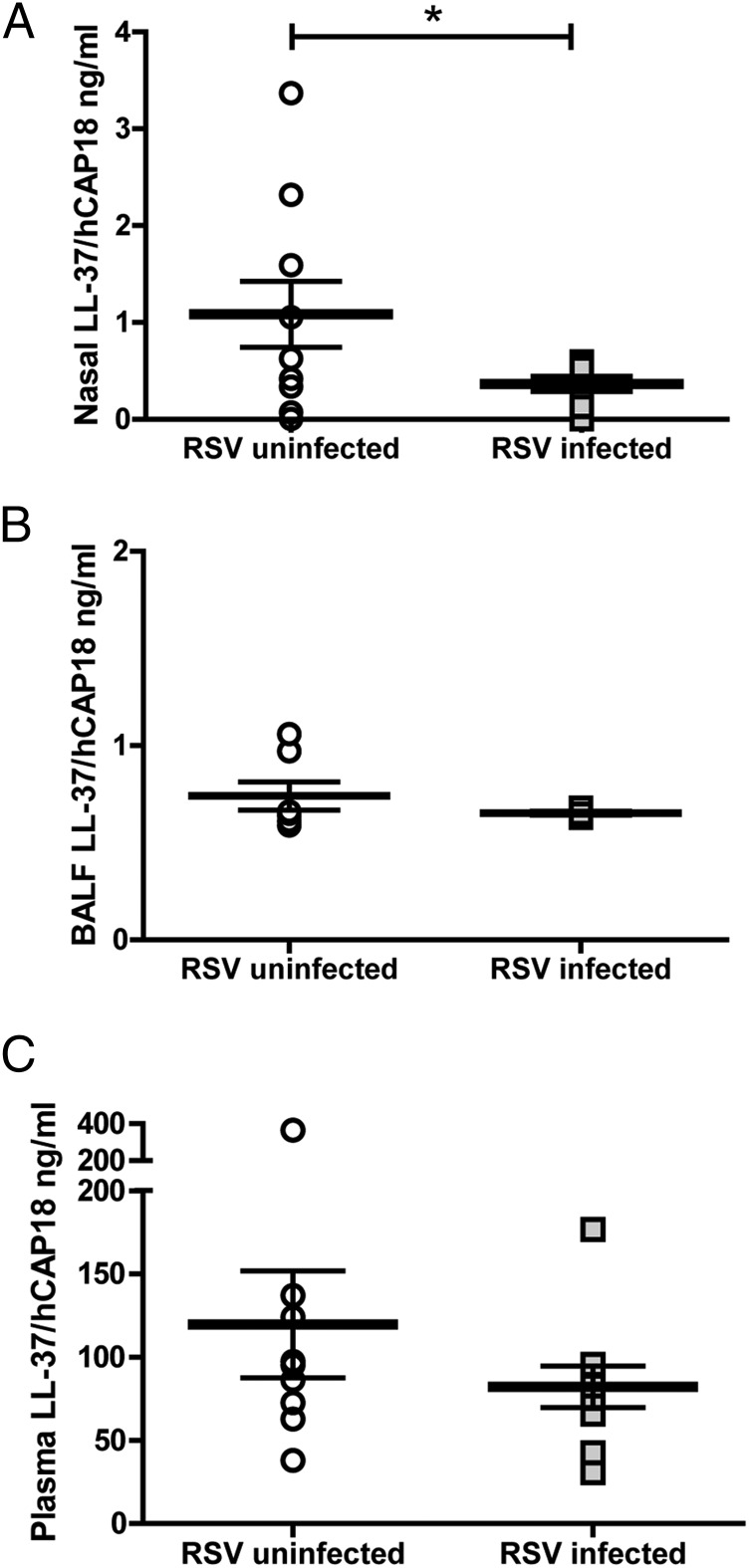
Higher nasal LL-37 levels are associated with protection in an experimental human RSV infection model. Nasal lavage (**A**), bronchoalveolar lavage fluid (**B**), and plasma (**C**) were collected from a cohort of healthy, nonsmoking adults (age 18–33 y), before intranasal inoculation with 10^4^ PFU RSV A Memphis 37. Individuals were monitored and categorized as RSV infected or RSV uninfected based on detection of nasal RSV by Q-RT-PCR on at least 2 d between days 2 and 10 postinfection. Nasal and plasma samples from 10 uninfected and 9 infected individuals, and bronchoalveolar lavage fluid samples from 7 uninfected and 5 infected individuals were quantified for LL-37/hCAP-18 levels by ELISA, in duplicate, in blinded fashion. Results are displayed as mean ± SEM and analyzed by *t* test, *p < 0.05.

The observations that endogenous cathelicidin expression had protective function against pulmonary RSV in mice in vivo, and that immediate interaction with LL-37 diminished the infectivity of RSV in vitro and in vivo, led to the hypothesis that higher constitutive, upper airway hCAP-18/LL-37 expression at the primary sites of infection with RSV may represent a protective barrier in humans in vivo. To investigate this hypothesis, we collected nasal lavage, bronchial lavage, and plasma samples from healthy adult volunteers before undergoing experimental challenge with RSV and assessed them for hCAP-18/LL-37 content, blinded to clinical outcome. A proportion of individuals, inoculated with RSV A Memphis 37 by intranasal drops, subsequently developed upper respiratory tract infection, based on detection of RSV by Q-RT-PCR in nasal lavage on at least 2 d between days 2 and 10 after inoculation. RSV-uninfected individuals were found to have had significantly greater levels of hCAP-18/LL-37 in their nasal lavages at day 0 (before RSV inoculation) than RSV-infected individuals (Fig. 9A). These differences were not recapitulated in bronchoalveolar lavage samples (Fig. 9B), nor were any significant differences observed in plasma cathelicidin levels (Fig. 9C). These data suggest that low constitutive nasal expression of hCAP-18/LL-37 at the point of contact with RSV may be associated with an increased susceptibility to infection in humans.

## Discussion

Infection with RSV is one of the most common causes of respiratory tract illness in infants, especially in premature babies, although it is also an important cause of morbidity and mortality in elderly and immunocompromised individuals (26–29). By the age of 2 y, almost every child has experienced RSV infection (30), resulting in severe, life-threatening disease in ∼2% of infants (2). National Health Service England recorded 5938 RSV-associated hospital admissions of infants in 2012–2013 (31), whereas globally, RSV infection results in an estimated 200,000 deaths in young children annually (3), with 3.4 million infants requiring hospitalization. Furthermore, studies suggest a correlation between RSV-triggered bronchiolitis and the risk for long-term health issues including recurrent wheezing, asthma, and allergic sensitization (4, 5, 32). Despite this, there are no effective, specific therapeutic interventions or vaccine.

In this study, we demonstrate that human and murine cathelicidins, widely expressed host defense peptides, have direct antiviral effects on RSV in vitro, have protective effects against RSV infection in vivo in murine models, and are associated with protection against RSV infection in adult humans. Cathelicidins have a broad spectrum of functions, both directly antibacterial and modulatory. The human cathelicidin LL-37 has been shown to promote bacterial clearance in murine pulmonary infection models (33, 34), and *Camp^−/−^* mice have increased susceptibility to bacterial infection in a wide range of organs, including skin (17), intestinal tract (35), urinary tract (36), cornea (37), and lung (34, 38), demonstrating the critical, nonredundant role for cathelicidin in murine host defense against bacterial infection. However, in addition to their antibacterial functions, some host defense peptides have also been shown to have various antiviral properties in vitro (39), an observation that we and others have recently extended to cathelicidins (7, 40, 41). However, the relative importance of directly microbicidal properties versus modulatory functions in the protective in vivo effects of these peptides against bacterial and viral infections remains to be determined.

The modulatory properties of cathelicidins include the capacity to modify pattern recognition receptor signaling, and, with specific relevance to viral infections, LL-37 has been shown to enhance viral dsRNA signaling via TLR3, promote cytokine responses in rhinovirus-infected human airway epithelial cells (42), and augment IFN-β responses of human keratinocytes upon stimulation with polyinosinic-polycytidylic acid (43). TLR3- and RIG-I–mediated type I IFN responses constitute a critical component of the antiviral host response to RSV (44), generating the hypothesis that the antiviral effects of LL-37 against RSV may be mediated via an upregulation of this system. However, our data demonstrated the opposite effect, with LL-37 coadministration leading to significantly lower production of type I and type III IFNs by HEp-2 cells in vitro. A recent study demonstrated that alveolar macrophages are the key type I IFN-producing cells in murine RSV infection in vivo (45), raising the possibility that this LL-37–mediated modulation of this system could remain relevant in vivo. However, our in vivo studies also demonstrated lower IFN-β induction by RSV in the presence of LL-37. These observations, in conjunction with our previous findings (7), confirm that the antiviral effects of LL-37 are not mediated through modulation of IFN pathways. Given the antiviral effects of cathelicidins, the lower levels of IFNs are instead compatible with direct effects of LL-37 on RSV lowering the effective infectious dose.

In vitro analysis of the interaction between LL-37 and RSV demonstrated direct binding of the virus by the peptide, disruption of viral particles, and decreased binding of RSV to epithelial cells in vitro. These data all suggest that the primary antiviral mechanism of the peptide in vitro resulted from direct activity on the viral particle. This was confirmed in vivo, with concomitant delivery of LL-37 required for maximally effective protection against disease and lower viral loads in our murine model. Intranasal instillation of LL-37 at 1 h before infection was equally effective at preventing early-phase weight loss, proposed to be triggered by very high initial viral load causing perivascular edema and damage of the airway epithelium (24). This is compatible with the concept of cathelicidin providing a first contact antiviral shield in the nose, diminishing early viral load. The observation that this did not result in effective longer-term protection against disease may reflect some loss of available LL-37 before infection, because of uptake by epithelial cells (46), or lack of colocalization of the two separate intranasal inocula, resulting in survival of a sufficient proportion of RSV to cause later disease. A primary directly antiviral mode of action for LL-37 is in keeping with the in vitro antiviral mechanisms described for LL-37 on influenza (41) and vaccinia virus (47) and human β-defensin 2 on RSV (19), for which viral membrane disruption are described. However, to date, evidence for significance of these antiviral properties of host defense peptides in vivo is minimal.

*Camp^−/−^* mice have been shown to develop significantly more pox skin lesions than controls postinfection with vaccinia virus (48), and we have previously demonstrated that inhaled LL-37 and mCRAMP can protect against influenza as effectively as zanamivir (a neuraminidase inhibitor currently used in humans) in a murine model (40). Our data now demonstrate that the intranasal delivery of exogenous cathelicidin is protective against RSV infection in vivo, but also, importantly, that *Camp^−/−^* mice develop significantly more severe disease than wild-type controls in response to infection with RSV. These data highlight a nonredundant role for cathelicidins in the natural host defense against this respiratory pathogen. The early-phase response to RSV infection was not affected in *Camp^−/−^* mice, an observation likely to be attributable to the low level of pulmonary expression of mCRAMP and absence of the cleaved, active peptide at the time of infection. This could represent an equivalent absence, in both genotypes, of a first point of contact antiviral shield (enhanced by prior or concomitant instillation of exogenous LL-37 in the other studies). However, we demonstrate that RSV infection induced pulmonary cathelicidin expression in wild type mice in vivo, compatible with previous in vitro studies (49), and that *Camp^−/−^* mice developed significantly greater later-phase weight loss, viral load, and lung inflammation. These data demonstrate the antiviral importance of endogenous cathelicidin expression. However, a role for modulatory properties of this peptide protecting against systemic disease cannot be excluded, irrespective of early virucidal properties. Although cathelicidin treatments significantly enhanced survival rates, and resulted in less weight loss and lower levels of inflammatory lung cytokines in influenza-infected mice, relatively minor reductions in viral load were observed (40). In addition, mice deficient in murine β-defensin 1 had significantly greater weight loss and lethality when infected with influenza, despite viral load being unaffected (50). Furthermore, we recently provided the first in vivo evidence of cathelicidin-mediated protection against infection acting primarily via inflammomodulatory properties, in a *Pseudomonas aeruginosa* murine lung infection model (34). Therefore, a detailed future examination of the impact of cathelicidins on the inflammatory and immunological responses to viruses, including to RSV, will be required to examine this possibility.

Our observations in *Camp^−/−^* mice demonstrate the importance of endogenous cathelicidin induced postinfection and, therefore, contrast with the absence of any protective effect of intranasal delivery of LL-37 given 1 h after RSV infection and daily thereafter. This may relate to inappropriate pulmonary localization of exogenously delivered peptide, as opposed to localized endogenous production, differences between LL-37 and mCRAMP, timing of deliveries, or localized extracellular or intracellular peptide concentrations. Regardless, these data suggest that delivery of cathelicidin, or synthetic analogs, could have therapeutic value in an established infection, if delivery and localization were correctly optimized. Furthermore, the direct antiviral effects of LL-37 in vitro and early-phase protection in vivo suggest the possible potential of interventional strategies aimed at enhancing this upper airways first point of contact antiviral shield. This approach is further supported by the observations from a human RSV experimental infection model that higher levels of nasal hCAP-18/LL-37 were associated with protection against RSV infection. It is important to recognize that this study evaluated infection in healthy adults, in whom RSV is not a clinical problem, and found that other mechanisms, such as IgA memory, are of significance to outcome in these cohorts (18). However, these data raise the possibility that baseline levels of cathelicidin in the upper respiratory tract may be of much greater significance in young infants with a first RSV infection, in whom adaptive immunity will not have the same significance in determining the outcome of infection. In that regard it will be important to study the factors that determine variations in baseline expression levels of cathelicidins.

Expression of cathelicidin in humans is regulated by vitamin D (51) and can be further enhanced by exposure to RSV in the presence of vitamin D (49). This raises the possibility that lower levels of vitamin D may impair cathelicidin-mediated defense against RSV. Lower serum 25(OH) vitamin D3 levels observed in winter are associated with increased risk for respiratory infections (52, 53), and newborns with low cord blood 25(OH) vitamin D3 levels have a significantly greater risk for subsequent RSV-associated lower respiratory tract infections (54). Median serum levels of hCAP-18/LL-37 in children with RSV bronchiolitis were found to be significantly lower than in those with human rhinovirus-induced bronchiolitis (55). Furthermore, RSV-infected children with hCAP-18/LL-37 levels lower than the median had more severe disease than those with hCAP-18/LL-37 levels above the median (55). In combination with our findings, these studies highlight the anti-RSV potential for therapeutic or preventative strategies that boost cathelicidin expression. The development of approaches for hCAP-18/LL-37 induction currently includes evaluation of the drug 4-phenylbutyrate, shown to upregulate cathelicidin with therapeutic potential in animal models (56–58) and/or the use of vitamin D supplementation, which should be considered as a novel preventative strategy against RSV infection, particularly in winter in the northern hemisphere.

Thus, our new data lead us to propose that cathelicidins are a key, nonredundant component of host defense against airway infection with RSV, functioning as a first point of contact antiviral shield, acting via peptide-mediated disruption of viral particles in the upper airways, and having additional later-phase roles in minimizing the severity of disease outcome. Consequently, cathelicidins represent an inducible target for preventative strategies against RSV infection and may inform the design of novel therapeutic analogs for use in established infection.
